# A new paradigm for defining trauma: bridging interdisciplinary perspectives

**DOI:** 10.3389/fpsyg.2026.1870152

**Published:** 2026-07-08

**Authors:** Sara Hirschorn, Jolanta Burke, Siobhán E. McCarthy

**Affiliations:** 1 Centre for Positive Health Sciences, RCSI University of Medicine & Health Sciences, Dublin, Ireland; 2 Graduate School for Healthcare Management, RCSI University of Medicine & Health Sciences, Dublin, Ireland

**Keywords:** trauma, DSM, PTSD, somatic paradigm, somatic trauma therapy, trauma definition

## 1 Introduction

Scholarly debate in psychology and psychiatry about how trauma should be defined is extensive and contentious, with varying and often directly conflicting stances regarding the most accurate and appropriate definition. Much of the disagreement centers around trauma's definition within posttraumatic stress disorder (PTSD) in the *Diagnostic and Statistical Manual for Mental Disorders* (DSM). Since PTSD's introduction to the DSM-III in 1980, symptoms listed under the disorder (within Criterion B) have evolved and currently include alterations in mood, cognition and arousal, as well as symptoms of intrusion and avoidance (American Psychiatric Association, 2022). However, the key point of disagreement centers on PTSD's Criterion A, which defines what type of stressors or events qualify as being traumatic. These events were first defined as those that “evoke significant symptoms of distress” (American Psychiatric Association, 1980), evolving in later editions of the DSM to those that are “markedly distressing” and “outside of the range of usual human experience” (American Psychiatric Association, 1987) (see Table 1). On subsequent recognition that traumatic experiences are relatively common, the latter definition was removed in the DSM-IV in 1994. Since then, Criterion A has explicitly specified events that qualify as traumatic: those that involve actual or threatened death, serious injury or sexual violence (American Psychiatric Association, 1994, 2000, 2013, 2022). However, debate continues, and a recent review identified four main and opposing positions: to expand criterion A, to narrow it, to eliminate it, or to keep it unchanged (Marx et al., 2024). The review ultimately resulted in a call for more research before any criterion adjustments are made. Despite 40 years of attempts to refine this aspect of PTSD's definition, controversy remains.

**Table 1:** Definitions of criterion A in Posttraumatic Stress Disorder (PTSD), by edition of Diagnostic and Statistical Manual of Mental Disorders (DSM).

DSM edition	Year	Criterion A definition
DSM-III^a^	1980	The existence of a recognizable stressor that would evoke significant symptoms of distress in almost everyone.
DSM-III-R^b^	1987	The person has experienced an event that is outside the range of usual human experience and that would be markedly distressing to almost anyone (e.g., serious threat to one's life or physical integrity; serious threat or harm to one's children, spouse, or other close relatives and friends; sudden destruction of one's home or community; or seeing another person who has recently been, or is being, seriously injured or killed as the result of an accident or physical violence.)
DSM-IV^c^	1994	The person has been exposed to a traumatic event in which both of the following were present: 1) The person experienced, witnessed or was confronted with an event or events that involved actual or threatened death or serious injury, or a threat to the physical integrity of self or others. 2) The person's response involved intense fear, helplessness, or horror.
DSM-IV-TR	2000	Identical to DSM-IV
DSM-5^d^	2013	A. Exposure to actual or threatened death, serious injury, or sexual violence in one or more of the following ways: 1. Directly experiencing the traumatic event(s). 2. Witnessing, in person, the event(s) as it occurred to others. 3. Learning that the traumatic event(s) occurred to a close family member or close friend. In cases of actual or threatened death of family member or friend, the event(s) must have been violent or accidental. 4. Experiencing repeated or extreme exposure to aversive details of the traumatic event(s) (e.g., first responders collecting human remains; police officers repeatedly exposed to details of child abuse). Note: Criterion A4 does not apply to exposure through electronic media, television, movies, or pictures, unless this exposure is work related.
DSM-5-TR	2022	Identical to DSM-5

^a^Reprinted with permission from the *Diagnostic and Statistical Manual of Mental Disorders* (3rd ed.). Copyright 1980 by the American Psychiatric Association.

^b^Reprinted with permission from the *Diagnostic and Statistical Manual of Mental Disorders* (3rd ed., text rev.). Copyright 1987 by the American Psychiatric Association.

^c^Reprinted with permission from the *Diagnostic and Statistical Manual of Mental Disorders* (4th ed., text rev.). Copyright 1994 by the American Psychiatric Association.

^d^Reprinted with permission from the *Diagnostic and Statistical Manual of Mental Disorders* (5th ed.). Copyright 2013 by the American Psychiatric Association.

**Figure 1:**
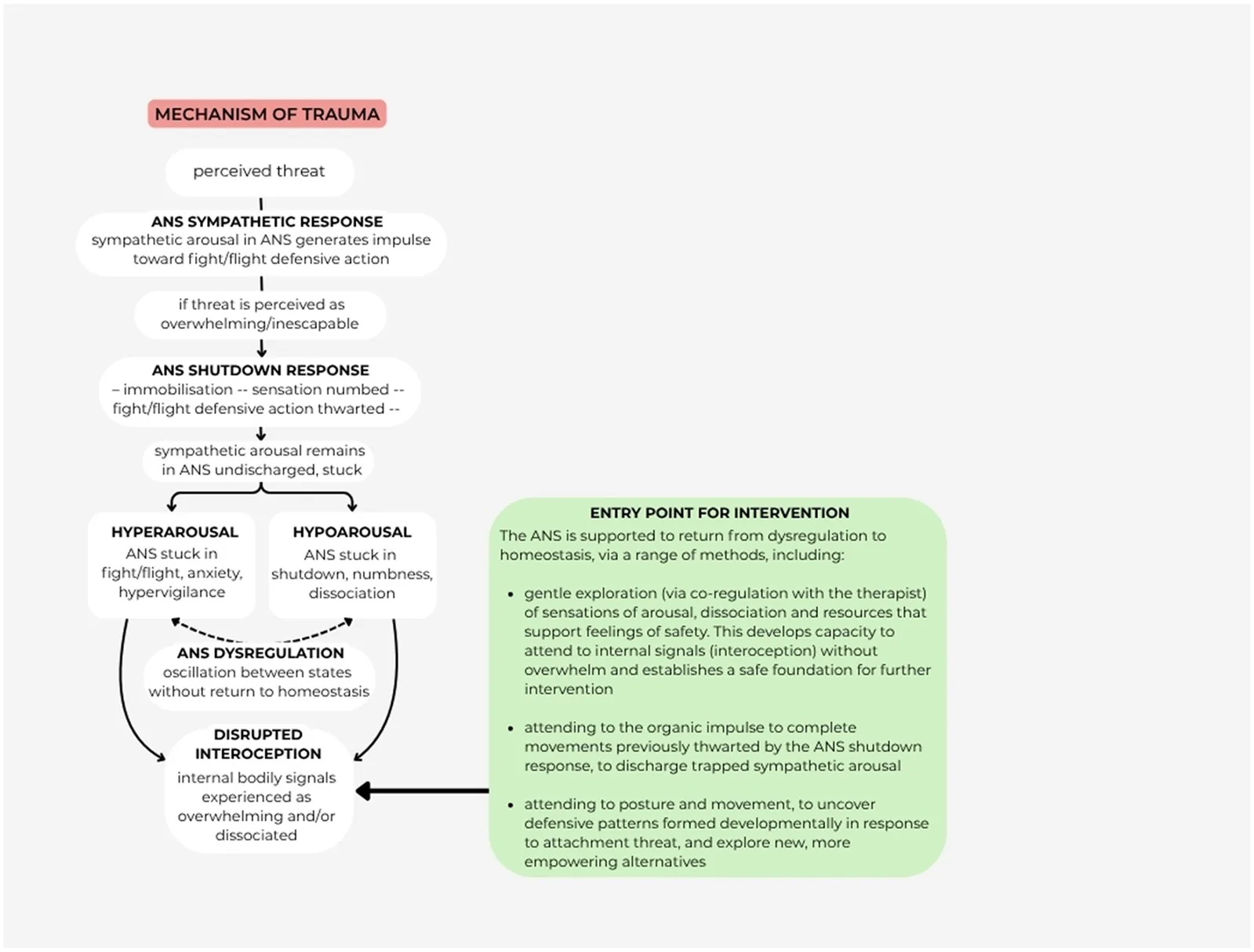
The mechanism of trauma and intervention in movement-based somatic trauma therapies.

Meanwhile, in the interdisciplinary field of somatic trauma therapy, a different conceptualization of trauma is proposed which can offer new perspective to this debate. There are multiple therapy modalities within the field, incorporating embodied aspects such as interoception, movement and touch, but sharing a common conceptualization of trauma; it is defined only by the physiological impact upon the person, rather than by aspects related to the event. Trauma is understood to cause dysregulation in the autonomic nervous system (ANS) and accordingly, resolution of post-trauma symptoms is sought by working directly with the body, using “bottom-up” approaches (Grassmann et al., 2025). For example, in Somatic Experiencing (SE; Levine, 1997), trauma is stated to occur when overwhelming sympathetic arousal activates a “shut down” response in the ANS (to numb the experience), which traps the sympathetic charge and causes dysregulation. SE's corresponding intervention purports to resolve symptoms by developing attention to internal sensations and impulses and completing movement patterns associated with the original fight/flight arousal that were thwarted by the “shut down” response. This mobilizes the trapped sympathetic charge and stuck patterns of hyper-arousal and/or dissociation, returning the ANS to homeostasis (Payne et al., 2015; Dann, 2024). Similarly, in Sensorimotor Psychotherapy (Ogden et al., 2006), trauma is posited to be encoded developmentally in postures, bodily sensations and movement patterns. Via processes of interoception and adjustment of defensive developmental embodied habits and movements, a return to homeostasis is enabled (see Figure 1). A range of other somatic modalities offer nuanced embodied approaches to restoring autonomic regulation, but it is their shared shift away from defining trauma in terms of the event, combined with recognition for the role of the body in trauma, that offers potential to alleviate some key points of conflict in the current debate in psychology and psychiatry.

Before outlining this potential contribution, it's important to note that despite decades' long practice, current evidence for the efficacy of somatic trauma therapies is only emergent, which may explain why the somatic conceptualization of trauma has not featured greatly in the debate to date. Accounting for this limited data however, is an evolving methodological critique highlighting the poor fit between complex experiential PTSD treatments (such as somatic trauma therapies), and conventional methodology for intervention validation, i.e., the randomized control trial (RCT) (Smith, 2024). While somatic therapy techniques are well-defined, they inherently adapt to the unique, complex and non-linear process of clients' post-trauma recovery, making them difficult to standardize (practically and ethically) for research purposes. This has led to a preference in the field for case studies (Kalisvaart and Ogden, 2025) and potential empirical under-representation of the range of clinically observed changes post-intervention, such as shifts in body awareness, self-reported regulation, resilience, vitality and relational safety (Heller and LaPierre, 2012; Holt and McLean, 2021; Kuhfuß et al., 2021). Smith (2024) cautions that given the complex nature of PTSD, sole reliance on the RCT in PTSD research may actively hinder capacity to guide practice, and calls for inclusion of qualitative, process-oriented and practice-based studies better suited to more complex interventions. This approach would enable a more holistic understanding of the full impact of somatic trauma interventions and represents an important avenue for future research.

Despite these methodological obstacles, evidence for the efficacy of somatic trauma interventions from controlled and uncontrolled studies (including the RCT) is promising, demonstrating clinically meaningful reductions in PTSD and related symptoms (Gene-Cos et al., 2016; Classen et al., 2020; Kuhfuß et al., 2021; da Silva et al., 2025; Pelixo et al., 2025). Something not definitively evidenced, is the mechanism by which somatic trauma therapies effect change. While enhanced autonomic regulation has been documented via self-report (Kuhfuß et al., 2021), research utilizing direct biomarkers of autonomic change is a key area for further empirical study. Overall, however, the somatic paradigm's contention that trauma disrupts autonomic regulation, alters interoception and leaves the ANS stuck in patterns of hyperarousal and “shut down” is supported by a growing body of evidence that interoceptive and autonomic dysregulation play a mechanistic role in post-traumatic symptomology (e.g., Williamson et al., 2015; Soder et al., 2019; Joshi et al., 2023; Leech et al., 2024). This increasing evidence for the role of the body and nervous system in trauma and trauma therapy, which has typically been absent within the psychiatric model, supports the contention of a “paradigm shift” in the field (Riordan et al., 2019) and again underlines why further empirical exploration is warranted in this domain.

## 2 Useful aspects of the somatic paradigm for the trauma definition debate

### 2.1 Potential for enhanced objectivity in trauma diagnoses

A criticism of the DSM's current framing of PTSD is that systemic and cultural bias is inherent in definitions of events that qualify as traumatic (and therefore who qualifies for trauma-related healthcare). For example, insidious systemic trauma to marginalized individuals (e.g., social displacement and oppression) has been rendered “invisible” (Saraiya et al., 2025). A similar lack of visibility is noted for chronic and developmental traumas, which may not be based on identifiable or catastrophic events (Bureau et al., 2010). For example, the chronic and seemingly ordinary experience of early parental misattunement, whereby the parent/caregiver is not reliably responsive to an infant's expressed emotions, is a well-documented cause of emotional dysregulation and long-term physiological and psychological harm (Schore, 2009; Farina and Schimmenti, 2025), but is not acknowledged in current psychiatric definitions and rendered a “hidden trauma” (Bureau et al., 2010). Here the somatic paradigm, by shifting the definition of trauma toward the physiological response rather than event characteristics, can offer visibility to such traumas, reducing the risk of subjective and cultural bias.

Offering potential for further objectivity in trauma diagnoses, the physiological underpinning of the somatic paradigm lends itself to the use of biomarkers as indices for nervous system dysregulation caused by trauma. While biomarker research in trauma is still emergent, indices such as heart rate variability and respiratory sinus arrhythmia show promising utility to this end, particularly within a multi-modal approach (Campbell et al., 2019; Guichard et al., 2024). As research in this area evolves, potential grows for increased objectivity in trauma diagnoses, supporting a move away from subjective definitions of trauma and the risk of bias they present.

### 2.2 Conceptual alignment with calls for reform in psychiatric nosology

Clark et al. (2017) note thatthe DSM's original purpose of statistical measurement has led to a false conception of distinct mental disorders, when in fact evidence demonstrates high comorbidity between diagnoses, which themselves are dimensional. This critique is consistent with the DSM-5′s own acknowledgment that its categories may not reflect clinical reality and its call for dimensional approaches that cut across current categories (American Psychiatric Association, 2013). In place of the binary notion of a disorder either being present or not, Clark et al. (2017) call for a dimensional and transdiagnostic approach that more accurately represents the reality of symptom continua and mixed presentations. Additionally, Bransford and Blizard (2017) note the DSM's solely descriptive focus on symptoms does not acknowledge, outside of the PTSD criterion, the well-documented traumatic etiology underlying multiple disorders (Mauritz et al., 2013; Sunderland et al., 2016; Lewis and Danese, 2023). While further empirical research in the field is imperative, the foundations of the somatic paradigm offer conceptual alignment with the above calls for reform. A spectrum of trauma symptoms is hypothesized, ranging from clinical to more subtle manifestations, such as emotional numbness and somatic complaints (Levine, 1997; Fisher, 2019). Further, autonomic dysregulation from trauma is proposed to have transdiagnostic impact, underlying a range of disorders such as mood disorders, anxiety disorders and borderline personality disorder (Fisher, 2019; Maté and Maté, 2022). Based on decades of clinical observation, the somatic paradigm's dimensional and transdiagnostic positioning resonates with current calls for nosological reform and warrants further empirical exploration.

Such exploration may have particular merit, given the potential implications of the somatic paradigm's hypothesis that a wider range of traumatizing events and symptoms exists than current psychiatric nosology acknowledges (Levine, 1997; Fisher, 2019). Conceptually, trauma may be more nuanced, insidious and therefore prevalent, than currently understood. Clinically, effective trauma interventions (including somatic therapies) may have scope beyond psychiatric presentations to include sub-clinical symptomology. Empirical research exploring somatic trauma interventions in sub-clinical populations is currently limited, however, some initial evidence suggests their efficacy in significantly reducing anxiety and somatic symptoms in general and occupational populations (Winblad et al., 2018; Kuhfuß et al., 2021), lending support to the contention that their impact may extend beyond clinical populations and representing another key avenue for further research.

### 2.3 A destigmatising and resilience-building orientation

While the somatic conceptualization of trauma implies a wider range of events may be traumatizing than currently understood, calls to include a broader range of events within PTSD's criterion A have raised objections. Referred to as “concept creep” by critics, broadening criterion A has been deemed a pessimistic and potentially harmful proposition (McNally, 2010; Haslam and McGrath, 2024). Concerns include that it would place an overemphasis on pathology, medicalise minor events and therefore damage the resilience of individuals who experience them. However, each of these concerns could be alleviated if trauma is considered from the somatic perspective. Post-traumatic symptomology is not pathologised; somatic conceptualisations propose it reflects an adaptive ANS response to situations perceived as overwhelmingly threatening (Payne et al., 2015). Further, somatic interventions propose to restore ANS regulation, thereby building resilience (Ergos Institute, 2025), with emergent empirical support for this assertion (Winblad et al., 2018). As noted, further research is needed to definitively validate these contentions utilizing direct biomarkers to explore underlying mechanisms beyond self-report, and mixed-methods to discover the full impact of somatic interventions. However, as its starting point, to ease “concept creep” concerns, the somatic paradigm offers a notably optimistic and growth-oriented framework; symptomology is destigmatised (without medicalising) and interventions propose organic resolution of symptoms at their root (Levine, 1997; Heller and LaPierre, 2012; Fisher, 2019).

## 3 Conclusion

After more than 40 years of unresolved debate about trauma's definition in psychology and psychiatry, the somatic conceptualization of trauma and somatic interventions warrant inclusion in ongoing research to bridge existing interdisciplinary siloes. The somatic conceptualization of trauma aligns with the call for a transdiagnostic and dimensional approach to mental disorder (American Psychiatric Association, 2013; Clark et al., 2017) that may enable further understanding of the mechanisms of post-traumatic symptomology and the etiology of a range of disorders. Its conceptualization also minimizes risks of subjective bias in identifying trauma and offers visibility to chronic and systemic traumas that are overlooked by current definitions.

Further, the contention that trauma's manifestation may include broader sub-clinical presentations, warrants empirical exploration. Validation of this proposition would imply clinical relevance both in psychiatric populations and a wider sub-clinical demographic, with potential scope to relieve suffering and improve wellbeing in broader sections of the general population.
