# Correction: A new paradigm for defining trauma: bridging interdisciplinary perspectives

**DOI:** 10.3389/fpsyg.2026.2001943

**Published:** 2026-09-30

**Authors:** Sara Hirschorn, Jolanta Burke, Siobhán E. McCarthy

**Affiliations:** 1 Centre for Positive Health Sciences, RCSI University of Medicine & Health Sciences, Dublin, Ireland; 2 Graduate School for Healthcare Management, RCSI University of Medicine & Health Sciences, Dublin, Ireland

**Keywords:** trauma, DSM, PTSD, somatic paradigm, somatic trauma therapy, trauma definition

An incorrect **Funding** statement was provided. The correct **Funding** statement reads:

The author(s) declared that financial support was received for this work and/or its publication. This publication has been developed within the framework of the Interreg North-West Europe 2021-2027 project Forest4Youth (project code NWE0400643), co-funded by the European Union.

The original version of this article has been updated.

